# Fhit Delocalizes Annexin A4 from Plasma Membrane to Cytosol and Sensitizes Lung Cancer Cells to Paclitaxel

**DOI:** 10.1371/journal.pone.0078610

**Published:** 2013-11-06

**Authors:** Eugenio Gaudio, Francesco Paduano, Riccardo Spizzo, Apollinaire Ngankeu, Nicola Zanesi, Marco Gaspari, Francesco Ortuso, Francesca Lovat, Jonathan Rock, Grace A. Hill, Mohamed Kaou, Giovanni Cuda, Rami I. Aqeilan, Stefano Alcaro, Carlo M. Croce, Francesco Trapasso

**Affiliations:** 1 Department of Molecular Immunology, Virology and Medical Genetics, The Ohio State University, Columbus, Ohio, United States of America; 2 Lymphoma and Genomics Research Program, IOR Institute of Oncology Research, Bellinzona, Switzerland; 3 Dipartimento di Medicina Sperimentale e Clinica, University Magna Græcia, Campus “S. Venuta”, Catanzaro, Italy; 4 Division of Experimental Oncology 2 CRO, Aviano, Italy; 5 Dipartimento di Scienze della Salute, University Magna Græcia, Campus “S. Venuta”, Catanzaro, Italy; 6 Department of Pathology, The Ohio State University, Columbus, Ohio, United States of America; 7 The Lautenberg Center for Immunology and Cancer Research, Institute for Medical Research, The Hebrew University, Jerusalem, Israel; university of Catanzaro, United States of America

## Abstract

Fhit protein is lost or reduced in a large fraction of human tumors, and its restoration triggers apoptosis and suppresses tumor formation or progression in preclinical models. Here, we describe the identification of candidate Fhit-interacting proteins with cytosolic and plasma membrane localization. Among these, Annexin 4 (ANXA4) was validated by co-immunoprecipitation and confocal microscopy as a partner of this novel Fhit protein complex. Here we report that overexpression of Fhit prevents Annexin A4 translocation from cytosol to plasma membrane in A549 lung cancer cells treated with paclitaxel. Moreover, paclitaxel administration in combination with Ad*FHIT* acts synergistically to increase the apoptotic rate of tumor cells both *in vitro* and *in vivo* experiments.

## Introduction

The *FHIT* gene, encompassing *FRA3B*, the most active common fragile site at chromosome 3p14.2 [1], [2] is a member of the histidine triad (HIT) family of proteins, encompassing a group of nucleotide-binding and hydrolyzing proteins with histidine triad motifs, represented in all organisms [3].

*FHIT* encodes a tumor suppressor protein whose loss is implicated in a large fraction of cancers; in fact, its deletion or loss of expression has been reported in head and neck [4], [5], gastrointestinal [6], cervical [7], lung [8], breast [9], [10], and hematopoietic tumors [11]. The role of Fhit in tumorigenesis was confirmed in mice, where *Fhit* genetic ablation resulted in the increased susceptibility to a wide spectrum of spontaneous tumors [12], [13]. Moreover, the deletion of either one or both *Fhit* alleles in mice enhanced sensitivity to the carcinogen N-nitrosomethylbenzylamine (NMBA), with most *Fhit^−/−^* and *Fhit* ^+/−^ mice developing forestomach tumors (adenomas, papillomas, and invasive carcinomas) after one dose of NMBA compared to wild-type controls [12], [14] which developed very few. Interestingly, *FHIT* was also successfully used as a therapeutic gene in cancer cell lines, where it triggers apoptosis, and several preclinical models of *FHIT*-null cancer, including lung, esophagus, pancreas, breast and leukemia [15]–[20]. However, reports concerning mechanisms of Fhit suppressor function have been more sparse and more recent. Fhit encodes a diadenosine polyphosphate (ApnA) hydrolase that cleaves substrates such as diadenosine *P* ^1^,*P* ^3^ triphosphate (ApppA) and diadenosine 5′,5′′′-*P* ^1^,*P* ^4^-tetraphosphate (AppppA) to AMP plus the other nucleotide [21], [22]. Through adenoviral expression of *FHIT* mutant alleles, we demonstrated that Fhit-substrate binding is limiting for tumor suppression, while its catalytic activity is not required for Fhit ability to trigger apoptosis [23]; moreover, we showed that the highly conserved Fhit tyrosine 114 (Y114), which can be phosphorylated by Src [24], is necessary to trigger the caspase-dependent Fhit-mediated apoptosis [25]. More recently, by using a proteomic approach, we identified a Fhit protein complex including Hsp60 and Hsp10, which may mediate both Fhit stability and its mitochondrial localization; once in the mitochondria, Fhit binds and stabilizes ferredoxin reductase (Fdxr), leading to modulation of the production of reactive oxygen species (ROS), an early step in Fhit-induced apoptosis [26]. The evidence of Fhit mitochondrial localization led also to the discovery that it increases the accumulation of Ca2+ into the organelle, in accord with its role in apoptosis under appropriate conditions [27].

The wide subcellular distribution of Fhit encouraged us to continue the search for novel Fhit protein partners to further shed light on its role in tumor suppression. Here, we demonstrate that Fhit interacts with Annexin 4; this interaction can block the translocation of Annexin 4 from cytosol to plasma membrane during treatment of lung cancer cells with paclitaxel. As a consequence, exogenous *FHIT* restoration in *FHIT*-null cancer cells acts synergistically with paclitaxel in triggering apoptosis *in vitro* and tumor regression *in vivo*. Taken together our findings suggest that Fhit restoration could have a role in overcoming drug resistance and that combination with paclitaxel can represents a valid approach for cancer therapy.

## Results

### Isolation of Fhit protein complexes from cell membranes

In our previous proteomic approach, we isolated a soluble Fhit protein complex from A549 cancer cells [26]. By using a slightly modified approach, aimed at identifying Fhit-interacting proteins in cell membranes, we used a recombinant adenovirus carrying a *FHIT* cDNA modified at its 3′ end with a sequence encoding a histidine-six epitope tag (Ad*FHIT-*His6), as previously described [26]. Briefly, A549 lung cancer cells were infected either with Ad*FHIT* (used as a control) or Ad*FHIT-*His6 at MOI50 and treated with dithiobis [succinimidylpropionate] (DSP), a cross-linker that crosses membranes and fixes proteins in complex in living cells. Cells were lysed and membrane enriched fraction isolated; Fhit-His6 recombinant protein along with its candidate protein partners was isolated with nickel beads avid for the His6 epitope tag. Purified proteins were treated with dithiothreitol (DTT) to cleave DSP and dissociate the complex, and digested by trypsin; protein components were identified by liquid/chromatography tandem mass spectrometry (LC-MS/MS). After protein identification by database search, inspection of LC-MS/MS data was undertaken to assess exclusive presence of mass peaks belonging to candidate partner proteins in samples from cells infected with Ad*FHIT-*His6. Proteins identified are listed in Table 1.

**Table 1:** List of putative interactors of Fhit protein identified by mass spectrometry.

Protein	Accession no. in NCBI/HUGO gene name	Mr KDa	Subcellular localization	Peptide Sequences	Individual peptides score	Mascot score
Aldehyde dehydrogenase	.gi/2183299 ALDH1	55.4	Mitochondria	K.LADLIER.	37	238
				K.SLDDVIKR.A	37	
				K.RVTLELGGK.S	42	
				K.VAFTGSTEVGK.L	66	
				K.ILDLIESGKK.E	62	
				K.EEIFGPVQQIMK.F	86	
				R.TIPIDGNFFTYTR.H	43	
				R.ANNTFYGLSAGVFTK.D	7	
				R.IFVEESIYDEFVR.R	55	
				K.AVKAARQAFQIGSPWR.T	26	
				K.LECGGGPWGNKGYFVQPTVFSNVTDEMR.I	108	
2-phosphopyruvate-hydratase alpha enolase	.gi/693933 ENO1L1	47.4	Cytosol	K.LAQANGWGVMVSHR.S	27	122
				R.AAVPSGASTGIYEALELR.D	122	
Elongation factor 2	.gi/31108 EEF2	96.2	Cytosol	M.VNFTVDQIR.A	25	110
				K.EGIPALDNFLDKL.-	77	
				R.KIWCFGPDGTGPNILTDITK.G	18	
Mitochondrial malate dehydrogenase	.gi/12804929 MDH2	36	Mitochondria	K.VAVLGASGGIGQPLSLLLK.N	109	109
Tubulin 5beta	.gi/35959 TUBB4A	50	Cytosol	R.SGPFGQIFRPDNFVFGQSGAGNNWAK.G	80	94
Annexin A2	.gi/16306978 ANXA2	38.8	Cytosol/cell membrane	R.DALNIETAIK.T	45	88
				R.QDIAFAYQR.R	46	
				R.SNAQRQDIAFAYQR.R	22	
				K.LSLEGDHSTPPSAYGSVK.A	26	
				R.RAEDGSVIDYELIDQDAR.D	69	
Annexin IV (PP4-X)	.gi/189617 ANXA4	36.3	Cytosol/cell membrane	R.DEGNYLDDALVR.Q	56	68
				R.QDAQDLYEAGEKK.W	19	
				K.SETSGSFEDALLAIVK.C	19	
				K.GLGTDEDAIISVLAYR.N	40	
Ribosomal protein L7a	.gi/4506661 RPL7A	30.1	Ribosome	R.LKVPPAINQFTQALDR.Q	66	66
Pyruvate kinase	.gi/35505 PKM2	58.4	Cytosol	K.FGVEQDVDMVFASFIRK.A	13	62
				R.TATESFASDPILYRPVAVALDTK.G	62	
Peroxidoxin 1	.gi/32455264 PRDX1	22.3	Cytosol	R.QITVNDLPVGR.S	53	59
Heat shock 90 kDa protein	.gi/306891 HSP90	83.6	Cytosol	R.GVVDSEDLPLNISR.E	31	50
				K.SLTNDWEDHLAVK.H	46	
				R.YESLTDPSKLDSGK.E	12	

### Fhit protein interacts with Annexin 4

It was previously demonstrated that the absence of Fhit protein in cancer cells resulted in resistance to paclitaxel [28]; Annexin 4, a protein with both plasma membrane and cytoplasmic localization, has been reported to be overexpressed in many cancer cells; in particular, it has been demonstrated that Annexin 4 over-expression contributes to cancer cells resistant to paclitaxel [29]. Thus, to shed light on the mechanisms of drug resistance in *FHIT*-minus cancer cells, we decided to focus on Annexin 4 among the freshly identified candidate Fhit partners. Annexin 4 belongs to a super-family of closely related calcium- and membrane-binding proteins that participate in a variety of cellular functions, including vesicle trafficking, cell division, apoptosis, calcium signalling and growth regulation. It has been proposed that some changes in annexin expression during tumourigenesis may result in resistance to chemotherapeutic agents and that individual annexins may prove to be therapeutic targets [30]–[35]. The Annexin 4 (ANX4) gene encodes a protein (namely, ANX4 or A4) almost exclusively expressed in epithelial cells [31] and overexpressed during paclitaxel treatment and in paclitaxel-resistant cell lines; transfection of ANX4 cDNA into 293 T cells resulted in a threefold increase in paclitaxel resistance [29], [36]. A correlation between the presence of Annexin 1 (ANX1) and MDR (multi drug resistance) proteins in breast cancer cells, provided the first direct evidence for a role of an annexin in multidrug resistance [37] .

**Figure 1:**
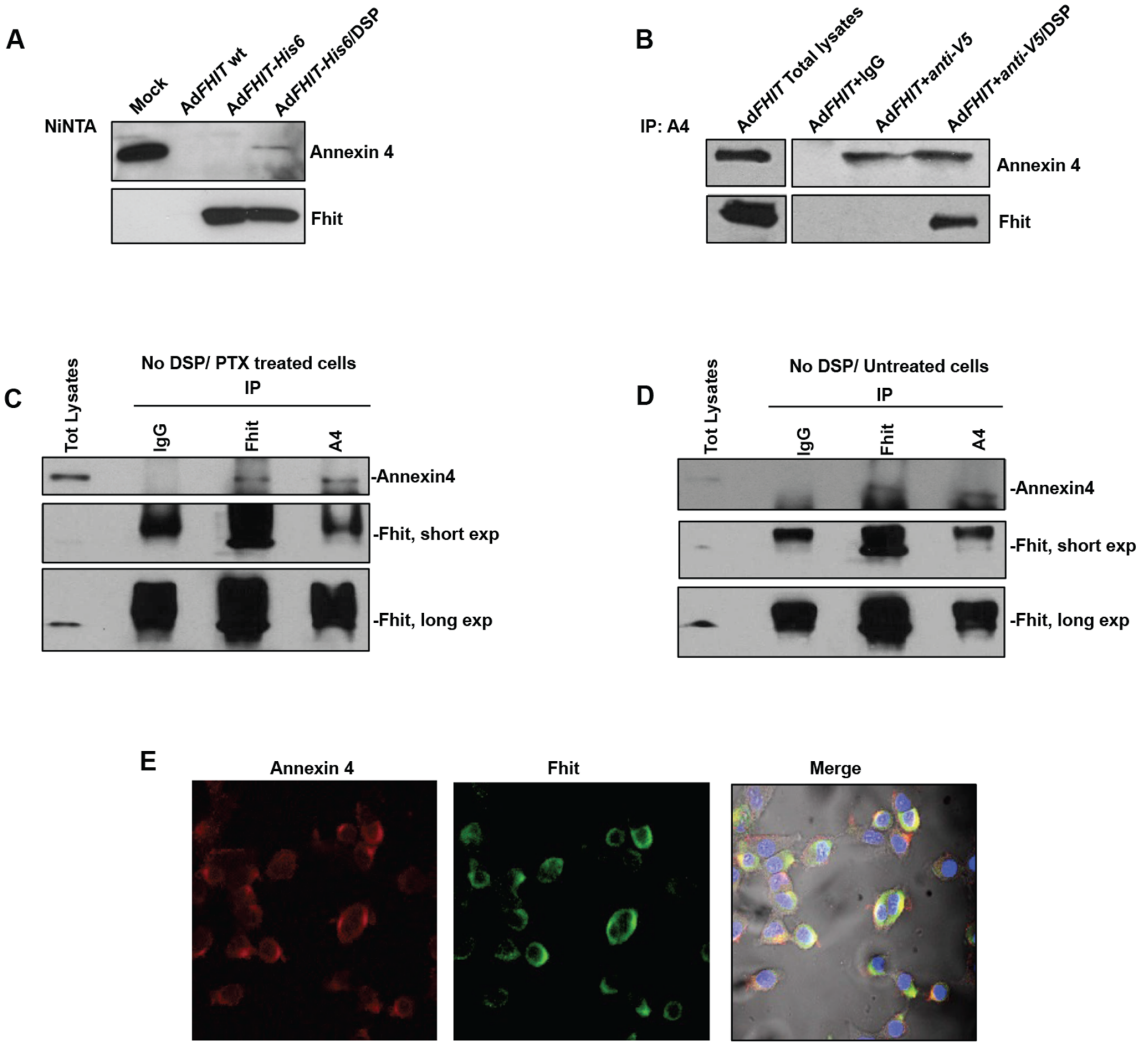
Fhit physically interacts with Annexin 4. **A**. A549 lung cancer cells were infected with Ad*FHIT*-wild type or Ad*FHIT-*His6 at MOI50; 48 h after infection, cells were treated with DSP and lysed with Mem-PER Eukaryotic Membrane Protein Extraction Kit to provide total lysates enriched in membrane fraction. Total lysates were immunoprecipitated with nickel beads. Immunoprecipitates were analyzed by immunoblotting (IB) with anti-Fhit and anti-Annexin A4 antibodies. **B**. A549 cells were transiently transfected with an expression plasmid encoding mammalian *Annexin4-*V5 (8 µg). 48 h after transfection, cells were treated with DSP and total lysates were immunoprecipitated (IP) with anti-V5 antibody. The immunoprecipitates were probed by immunoblotting (IB) with anti-Fhit and anti-Annexin 4 antibodies. **C-D**. HEK293 cells were mock tretated or tretaed with paclitaxel for 24 hrs (800 nM) and lysed. Total protein lysates were Immunoprecipited with IgG, Fhit and Annexin 4 antibodies, as indicated. The detection of endogenous Annexin 4 and Fhit was performed without the use of the DSP cross-linker.

To assess Fhit and Annexin 4 interaction, we performed a co-immunoprecipitation experiment using protein lysates prepared as described above (Figure 1, A and B). Fhit and Annexin 4 colocalization was investigated by confocal microscopy. As shown in Figure 1E, both Fhit and Annexin 4 mainly show a cytoplasmic subcellular colocalization.

As shown in Figure 1A and 1B, Fhit and Annexin 4 interaction was mainly detected in cytosolic extracts. We further carried out co-immunoprecipitation experiments on endogenous Fhit and Annexin 4 proteins in HEK293 cells and we demonstrated the interaction between these proteins also in absence of the DSP cross-linker, either with or without paclitaxel (Figures 1C and 1D). The immunoprecipitation carried out with Fhit antibody followed by an immunoblotting performed with an Annexin 4 antiserum, was clearly suggestive of a Fhit-Annexin 4 endogenous complex in intact cells.The direct way of the IPs between endogenous proteins were clearly indicative of the interaction (IP: Fhit, and IB: Annexin 4). Unfortunately, because of technical difficulties due to the co-migration of IgG and Fhit protein, the reverse approach, that is the immunoprecipitation of endogenous Annexin 4 protein followed by Fhit detection, was unsuccessuful.

### Fhit overexpression blocks Annexin 4 translocation from cytosol to plasma membrane

**Figure 2:**
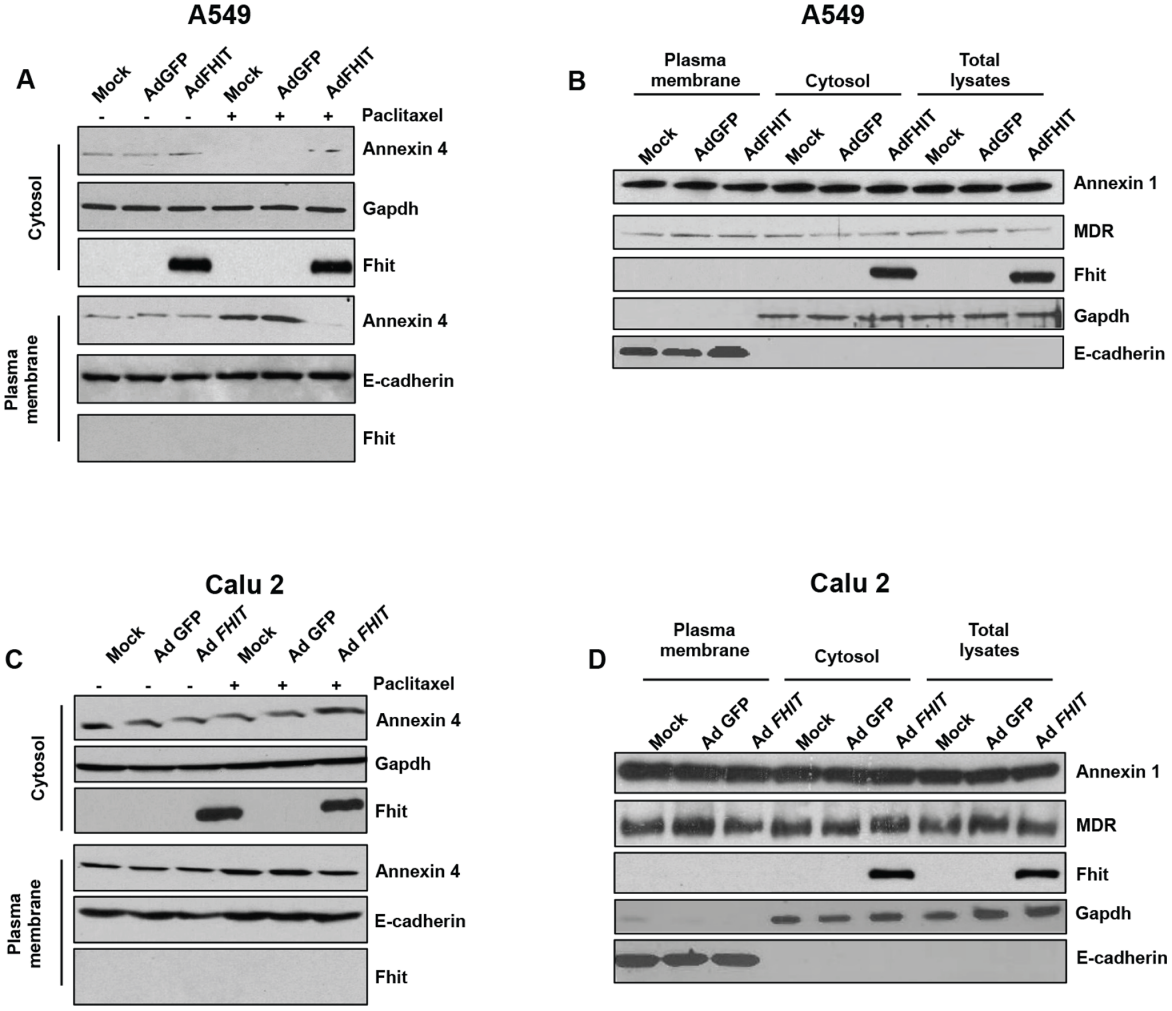
Fhit overexpression blocks Annexin 4 translocation from cytosol to plasma membrane. **A-C**. A549 and Calu-2 lung cancer cells were infected with Ad*GFP* or Ad*FHIT* at MOI50; 48 h later, cells were mock-treated or treated with paclitaxel (800 nM) for an additional 24 h. Proteins from cytosolic and membrane fractions were separated on a polyacrylamide gel, transferred to nitrocellulose filter, and probed with Annexin 4 antibody. Gapdh and E-cadherin were used to normalize protein loading of cytosolic and plasma membrane proteins, respectively. **B-D**. A549 and Calu-2 lung cancer cells were infected with Ad*GFP* or Ad*FHIT*-wild-type; 48 h later, cells were treated with paclitaxel (800 nM) for an additional 24 h. Proteins from cytosolic and membrane fractions were separated on a polyacrylamide gel, transferred to nitrocellulose filter, and probed with Annexin 1 and MDR (multi-drug resistance protein) antibodies. Gapdh and E-cadherin were used to normalize protein loading of cytosolic and plasma membrane proteins, respectively.

Fhit and Annexin 4 protein expression was evaluated in a panel of cancer cell lines (Figure S1), Fhit expression is lost in most cell lines investigated. To shed lights on the meaning of the Fhit-Annexin 4 protein complex, we performed our experiments on two of them, A549 and Calu-2, which show slight or no Fhit expression, respectively. To better define the subcellular localization of the Fhit-Annexin 4 protein complex, we performed cellular fractionation experiments. As shown in Figures 2A and 2C, basal Annexin 4 is distributed in both cytosol and plasma membrane. Treatment of A549 and Calu-2 lung cancer cells with paclitaxel induced cytosolic depletion of Annexin 4 that underwent an apparently complete translocation to the inner side of plasma membrane. Fhit overexpression blocked Annexin 4 translocation from cytosol to the plasma membrane, observed after paclitaxel administration. To show that the effects were specifically driven by the Fhit-Annexin 4 interaction, a similar experiment was performed considering the subcellular localization of Annexin 1 and MDR (multi-drug resistance protein), which are both involved in the resistance to chemotherapy [37], [38]. As shown in Figures 2B and 2D, neither Annexin I nor MDR subcellular localizations were influenced by Fhit overexpression. Taken together, these data indicate that Fhit protein specifically interferes with Annexin 4 translocation from cytosol to plasma membrane, observed in paclitaxel-treated A549 and Calu-2 cells.

### Annexin 4 depletion combined with Fhit overexpression and paclitaxel treatment synergistically induces proliferation inhibition and triggers apoptosis of lung cancer cells

**Figure 3:**
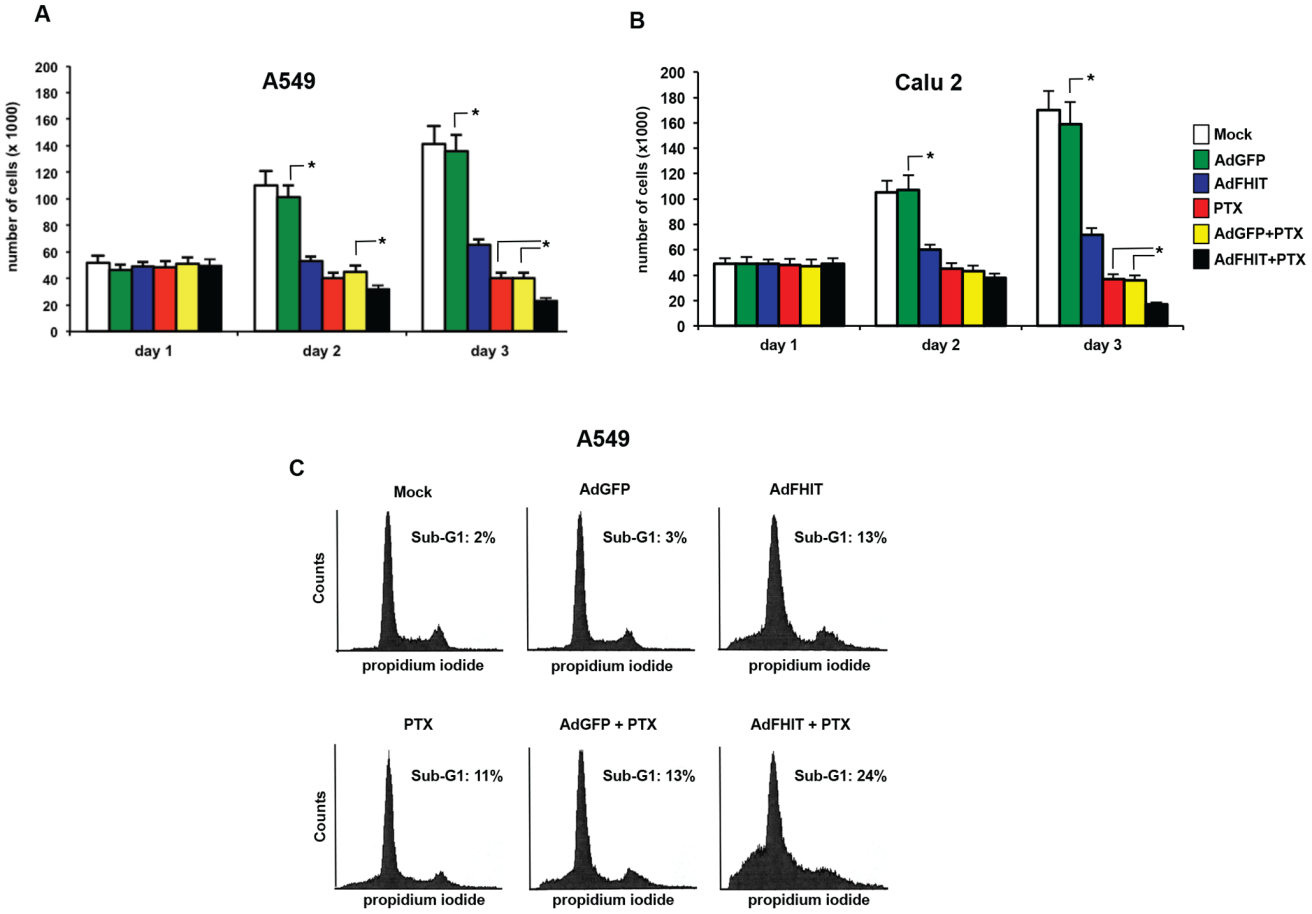
Fhit overexpression and paclitaxel treatment induces synergistic proliferation inhibition. **A-B**. A549 and Calu-2 cells were mock-infected, Ad*GFP* or Ad*FHIT* infected cells at MOI10 for 24 h, then treated with or without 800 nM paclitaxel for additional 6 h and counted. Bar graphs show mean ± SEM for values from three different experiments (* P<0,05). The Chou-Talalay methos was applied to calculate the nature of the combinations (CI<1, synergism). **C**. A549 cells were either mock-infected or infected with Ad*GFP* or Ad*FHIT*, or left untreated or treated with 800 nM paclitaxel, and then analyzed by flow cytometry.

To investigate the role of the Fhit-mediated block of Annexin 4 translocation from the cytosol to the plasma membrane in the mechanism of drug resistance, we performed cell growth curves under different conditions. As shown in Figure 3A and 3B for A549 and Calu-2 lung cancer cells, cell number was reduced in Ad*FHIT-*infected cells compared to controls; a slightly stronger cell growth inhibition was observed in cells treated with paclitaxel. Finally, consistent with previous reports [26], we observed a synergistic effect on the proliferation rate inhibition in A549 cells treated with paclitaxel plus Ad*FHIT*. In a representative experiment illustrated in Figure 3C, flow cytometry was used to investigate cell cycle perturbations in A549 cells infected with Ad*FHIT*, with (800 nM for 24 h) or without paclitaxel treatment; A549 cells infected with Ad*FHIT* showed a sub-G1 peak was detectable (13%), consistent with our previous findings [26]; a similar effect was obtained after 800 nM paclitaxel administration. A synergistic effect was detected after combining Ad*FHIT* and paclitaxel treatment (25% of sub-G1 fraction).

These data suggest that the restoration of Fhit function to Fhit-negative malignant cells, in combination with paclitaxel, might represent a potential novel approach for the treatment of patients affected by lung cancer. In fact, by lowering the threshold of sensitivity to drug administration in Annexin 4-mediated chemoresistant tumors, Fhit restoration might represent an intriguing approach to obtain a better patients outcome.

**Figure 4:**
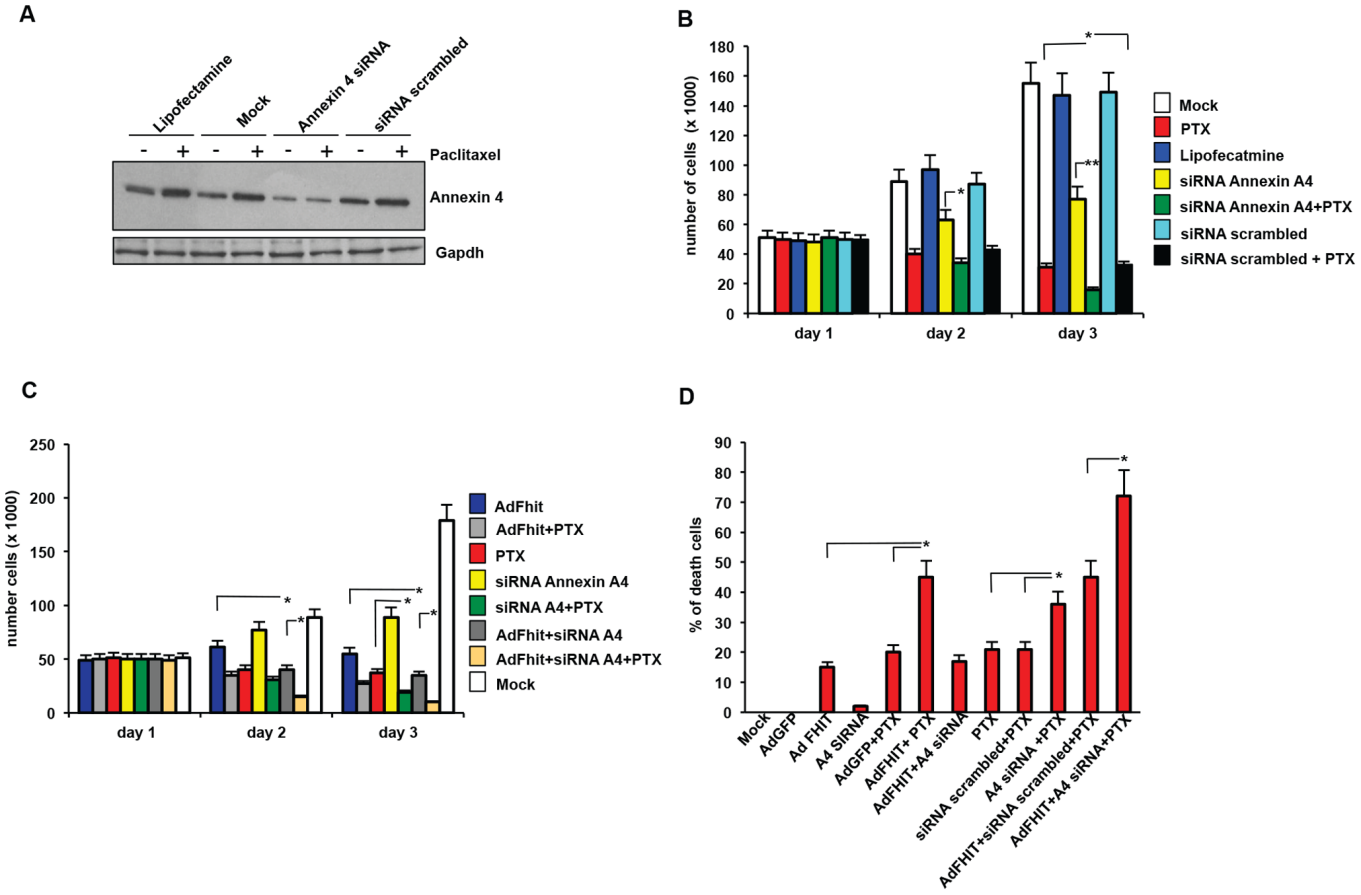
Annexin 4 depletion, combined with Fhit overexpression and paclitaxel treatment induces inhibition of proliferation and triggers apoptosis. A. A549 cells were mock-transfected or transfected with Annexin 4 siRNAs (50 nM) or scrambled siRNAs (50 nM) for 72 h. Cells lysates were immunoblotted with Annexin 4 and Gapdh antibodies. B. A549 cells were mock transfected or transfected with Annexin 4 siRNAs (50 nM) or scrambled siRNA (50 nM), infected with Ad*FHIT* at MOI25 for 72 h, and then left untreated or treated with paclitaxel (800 nM). Cells were first counted at 12 h after paclitaxel treatment. Bar graphs show mean ± SEM for values from 3 experiments (* P<0,05). The Chou-Talalay methos was applied to calculate the nature of the combinations (CI<1, synergism). C. A549 cells were mock transfected or transfected with Annexin 4 siRNA (50 nM) or scrambled siRNAs (50 nM) for 72 h, then untreated or treated with paclitaxel. Cells were first counted 12 h after paclitaxel treatment. Bar graphs show mean ± SEM for values from 3 experiments (* P<0,05). The Chou-Talalay methos was applied to calculate the nature of the combinations (CI<1, synergism). D. A549 cells, treated as described in B and C, were analyzed by TUNEL assay.

To determine if the interaction between Fhit and Annexin 4 could have a role on the Fhit-mediated cell growth inhibition and apoptosis, Annexin 4 was silenced with small interfering RNAs (siRNAs) designed to block Annexin 4 protein expression. As shown in Figure 4A, Annexin 4 protein expression levels were significantly reduced after Annexin 4 siRNAs transfection, in absence or presence of paclitaxel. Consistent with previous studies [36], mock-transfected cells and cells transfected with scrambled siRNAs showed Annexin 4 protein upregulation after paclitaxel administration compared to controls, whereas no changes were observed in A549 Annexin 4-depleted samples either in absence or presence of paclitaxel. The combination of paclitaxel treatment with Annexin 4 depletion had an synergistic effect on cell proliferation inhibition, with the cell number being significantly lower compared to the treatment with paclitaxel or Annexin 4-specific siRNA alone (Figure 4B). No effect on proliferation was observed after transfection with scrambled siRNAs. We also tested A549 cell proliferation rate after Annexin 4 silencing or infection with Ad-*FHIT* at MOI25 or combined treatments (Figure 4C). Annexin 4 depletion and Fhit overexpression caused a significant reduction in cell number compared to controls; a further dramatic reduction in cell number was observed by adding paclitaxel, indicating a synergistic effect.

A TUNEL assay confirmed that all sub-G1 populations described in Figure 3C were predominantly composed of apoptotic cells (Figure 4D). Moreover, also the effects on cell proliferation inhibition were paralleled by results of a TUNEL assay; in fact, apoptosis reached its highest extent in Annexin 4-depleted cells infected with Ad*FHIT* and treated with paclitaxel (Figure 4D).

### Fhit/Annexin 4 interaction plus paclitaxel induced tumor regression in a preclinical model of lung cancer

To investigate the effects of Fhit/Annexin A4 interaction *in vivo* with or without paclitaxel in a preclinical model of lung cancer, we performed an experiment on 11 groups of mice (n = 5 mice/group). Institutional Animal Care and Use Committee (IACUC) at the Ohio State University approved this study. Three groups were subcutaneously injected with 1×10^7^ A549 cells. When tumors reached 15 mm diameter, mice were mock-treated, treated with DMSO or treated with a single IV administration of 40 mg/kg paclitaxel; mice were monitored on a regular basis. Three days later, mice were sacrificed and tumors were evaluated by weight. Tumors from mice treated with paclitaxel showed a 50% reduction compared to controls. This short timepoint allowed us to magnify the effects of both single tretaments and their combination, as with longer timepoints the effects of the Fhit treatment would be covered by paclitaxel. Two groups of mice were injected with 1×10^7^ A549 cells pre-infected with Ad*GFP* or Ad*FHIT* at MOI50, and two more groups were injected with 1×10^7^ A549 cells pre-infected with Ad*FHIT* at low multiplicity of infection (MOI5); one of the latter groups was also treated with paclitaxel, as described above. Tumor weight showed 83% reduction compared to controls in mice injected with Ad*FHIT* MOI50 pre-infected cells versus 27% reduction in mice injected with Ad*FHIT* MOI5; interestingly, Ad*FHIT* MOI5 xenografts treated with paclitaxel showed tumor regression to 7% of control tumors, indicating a synergistic effect of Fhit and paclitaxel.

**Figure 5:**
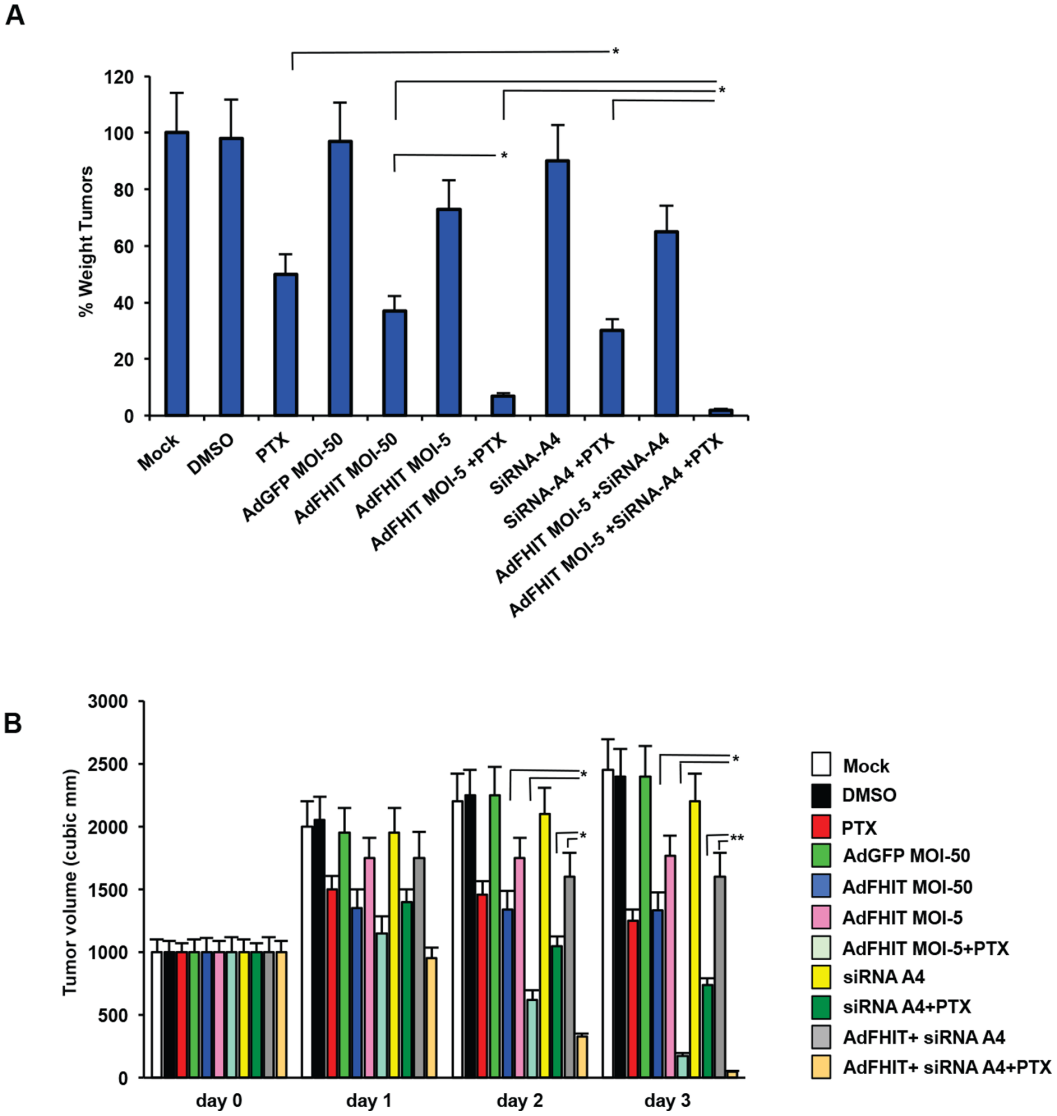
Fhit/Annexin 4 interaction plus paclitaxel induced tumor regression in a preclinical model of lung cancer. **A**. Nude mice were subcutaneously injected with 1×10^7^ A549 cells. Some groups (n = 5 mice/group) were injected with mock treated cells, others with cells transfected with Annexin 4 siRNA or infected with Ad*FHIT* (MOI5 or MOI50) or combinations of both. When tumors reached 15 mm diameter, mice were mock-treated, treated with DMSO or treated with a single IV administration of 40 mg/kg paclitaxel; mice were monitored on a regular basis. Three days after PTX, mice were sacrificed and tumors were evaluated by weight. Bar graphs show mean ± SEM for values from 5 mice (* P<0,05). The Chou-Talalay methos was applied to calculate the nature of the combinations (CI<1, synergism). **B**. Tumor volumes are reported over time.

To complete the panel, two groups of mice were injected with Annexin 4-depleted cells (one of which was treated with paclitaxel, as described above) and two more groups with Annexin 4-depleted cells pre-infected with Ad*FHIT* MOI5 (one treated with paclitaxel). Annexin 4-depleted cells showed a slight but not significant tumor reduction compared to controls, while a higher reduction was observed in Annexin 4-depleted xenografts treated with paclitaxel versus xenografts treated with paclitaxel alone (70% and 50%, respectively). Finally, virtually no tumors were detected in mice bearing xenografts with knocked-down Annexin 4 pre-infected with Ad*FHIT* MOI5 plus paclitaxel. Results of these experiments are reported in Figure 5A and 5B. Taken together, these data underline the importance of the Fhit/Annexin 4 interaction *in vivo* and highlight the value of combined Fhit gene therapy and chemotherapy in preclinical cancer models.

## Discussion

Loss of Fhit expression during tumorigenesis has been reported for most types of human cancer. Its role in malignant diseases is underlined by the fact that Fhit loss is an early event in tumors, as reported for lung precancerous lesions. Also, the successful *FHIT* gene therapy in a series of preclinical models of human cancer makes Fhit protein a good candidate for the search for innovative therapies [1]. Despite efforts to clarify how Fhit protein works, defining specific mechanisms through which Fhit modulates apoptotic and other tumor suppressor functions has been challenging. This was mainly due to the fact that, until a few years ago, Fhit had no confirmed protein partners identified by conventional studies aimed at isolation of protein-protein complexes. We have reported the isolation of Fhit-interacting proteins by a proteomics approach [26]; the investigation led to isolation and characterization of a soluble Fhit protein complex containing Hsp10/Hsp60 and ferredoxin reductase (Fdxr) among other mitochondrial proteins. In that study, we demonstrated that Hsp10/Hsp60 complex was involved in the translocation of Fhit protein into mitochondria, where its interaction with Fdxr was involved in modulation of production of reactive oxygen species, the earliest step in Fhit-mediated apoptosis. This approach, aimed at identification of protein complexes, suggested follow-up studies to identify other Fhit interactors and their functional signal pathways. In subcellular fractionation studies, Fhit protein was detected in all cell compartments but nucleus [26]. Thus, we isolated novel Fhit protein complexes from A549 protein lysates enriched in cell membranes. This approach produced a list of candidate Fhit partners, some of which were also identified in our previous analysis [26]. Among these new candidates, Annexin A4 (ANXA4) had been reported to play a role in drug resistance, with its expression increased in clones resistant to paclitaxel [36], a drug commonly used in the treatment of cancer patients. As we demonstrated in the past that Fhit itself is involved in overcoming paclitaxel resistance [26], [27], we decided to explore this potential correlation in further detail.

The interaction between Fhit and response to chemotherapy has been previously studied by Andriani et al [41]. In particular, Fhit expression resulted in reduced sensitivity to etoposide, doxorubicin, and topotecan. This feature was associated with Fhit-induced downregulation of DNA topoisomerases I and II. In contrast, Fhit expression produced a slight increase in sensitivity to cisplatin, as shown by colony-forming assays [41].

Inactivation of both FHIT and TP53 genes is frequently observed in primary non-small cell lung cancers (NSCLC) and cell lines and may contribute to resistance to apoptotic stimuli elicited by various anti-tumor drugs [42]. In this study, we demonstrated that paclitaxel administration induces both Annexin A4 up-regulation and modification of its intracellular distribution; in fact, following paclitaxel treatment, annexin A4 moves from cytosol to cell membrane. Interestingly, Fhit overexpression in Fhit-negative lung cancer cells prevents annexin A4 translocation from cytosol to cell membrane; furthermore, simultaneous treatment of A549 cancer cells with Ad*FHIT* and paclitaxel is much more effective in triggering apoptosis of cancer cells compared to controls; similar results were obtained with the administration of paclitaxel to annexin A4-depleted A549 cells. These data suggest that both annexin A4 overexpression and its plasma membrane subcellular localization in paclitaxel-resistant cancer cells is not a bystander effect but rather plays an active role in the mechanisms of drug resistance; further investigations of the function of annexin A4 in cell membrane might shed light on the complex mechanisms of drug resistance in cancer patients. These *in vitro* results found further support in a preclinical model of lung cancer; in fact, both *FHIT* gene therapy and annexin A4 depletion acted synergistically with paclitaxel in inducing tumor regression in mice compared to controls; this combination could be of useful interest in the treatment of lung cancer patients. In conclusion, previous and recent investigation underlines Fhit loss, a common feature in human cancer, as a marker of poor prognosis in cancer patients, suggesting that Fhit-negative tumors are prone to development of drug resistance.

## Materials and Methods

### Ethics Statement

Mice were maintained and animal experiments conducted under institutional guidelines established for the Animal Facility at The Ohio State University. This study was carried out in strict accordance with the recommendations in the Guide for the Care and Use of Laboratory Animals of the Ohio State University. The protocol was approved by the Institutional Animal Care and Use Committee (IACUC) at the Ohio State University (IACUC protocol number: 2010A00000146; approval date 8/15/2011). All surgery was performed under sodium pentobarbital anesthesia, and all efforts were made to minimize suffering.

### Cell culture and transfection experiments

A549 cells were maintained at 37° C in a humidified atmosphere of 5% CO_2_ in the appropriate growth medium with supplements added as recommended. HEK-293 cells were used for the generation and amplification of recombinant adenoviruses [18]. Transfections of Annexin 4 small interfering RNAs (Smart pool, Dharmacon) were carried out using Lipofectamine 2000 (Invitrogen).

### Immunoblotting and fractionation analysis

Total proteins were extracted with Nonidet P40 (NP-40) lysis buffer; cytosolic and plasmamembrane proteins were extracted using the FractionPREP-cell fractionation system (Biovision). Total lysates with enriched plasma membrane proteins, used for both mass spectometry and immunoprecipitations analyses, were obtained using Mem-PER Eukaryotic Membrane Protein Extraction Kit (Pierce).

For immunoblotting, proteins (50 µg) were separated on polyacrylamide gels and transferred to nitrocellulose filter membranes. Membranes were blocked in 5% non-fat dry milk, incubated with primary anti-Annexin 4, GAPDH, and E-Cadherin antibodies (Santa Cruz Biotechnology), detected by the appropriate secondary antibodies, and revealed by enhanced chemiluminescence (ECL; Amersham Inc.).

### Protein Interaction Analysis

Co-immunoprecipitation experiments, with or without dithiobis-[succinimidylpropionate] (DSP), a cross-linker from Pierce, were performed by incubating 1 mg of total protein lysates (containing enriched plasma membrane fraction) with either NIN-TA agarose magnetic nickel beads (Qiagen) or with anti-V5 antibody conjugated with Sepharose beads, overnight at 4°C; after washing, beads were boiled in 1×SDS sample buffer and proteins separated on 4–20% polyacrylamide gels (Bio-Rad).

### Immunofluorescence

A549 cells, pre-infected with Ad*FHIT*, were fixed in PFA 4% (paraformaldeyde) for 10 min, permeabilized 4 min with Triton 0.05% and analyzed by confocal microscopy. Endogenous Annexin 4 was detected with a primary antibody from BD; secondary antibody (594 nm) was from Molecular Probes.

### In vitro growth rate assessment

A549 cells were seeded at 1×10^5^ cells per 60-mm diameter dish and mock infected or infected with Ad*GFP* or Ad*FHIT* at MOI (Multiplicity of Infection) 50 and treated with 800 nM paclitaxel. Cells were monitored and counted at twenty-four h intervals after infection. Paclitaxel (LC-Laboratories) was dissolved in DMSO as a 1 mM stock solution.

### Generation of recombinant adenoviral vectors

Adenoviruses carrying *FHIT* or *FHIT-*His6 cDNAs (Ad*FHIT* and Ad*FHIT-*His6, respectively) under transcriptional control of a cytomegalovirus promoter were generated by homologous recombination in HEK-293 cells as previously described [26]. The Ad*GFP* vector was used as control.

### Protein Digestion

Immunoprecipitated protein complexes were digested with sequencing grade trypsin from Promega using the Multiscreen Solvinert Filter Plates from Millipore (Bedford). Briefly, the complexes were incubated with dithiothreitol (DTT) solution (25 mM in 100 mM ammonium bicarbonate) for 30 min prior to the addition of 55 mM iodoacetamide in 100 mM ammonium bicarbonate solution. Iodoacetamide was incubated with the protein-complexes in the dark for 30 min before removal. Enzymatic digestion was carried out with trypsin (12.5 ng/µL) for 18 h at 37°C. Digestion was stopped by adding 0.5% trifluoroacetic acid. The MS analysis was immediately performed to ensure high quality tryptic peptides with minimal non-specific peptides.

### Mass Spectrometry, LTQ and Protein identification

These studies were performed as previously described [39], [40].

### TUNEL assay

A549 cells were assessed for the induction of single strand breaks (indicative of apoptosis) by the terminal deoxynucleotidyl transferase mediated X-dUTP nick end labeling (TUNEL) assay using the *in situ* cell death detection kit (Boehringer/Roche), according to the manufacturer's recommendations.

### Flow cytometry

A549 cells were collected and washed in PBS solution. DNA was stained with propidium iodide (50 µg/ml) and analyzed with a FACScan flow cytometer (Becton-Dickinson) interfaced a Hewlett-Packard computer. Cell cycle data were analyzed with the CELL-FIT program (Becton-Dickinson).

### Animal studies

Mice were maintained and animal experiments conducted under institutional guidelines established for the Animal Facility at The Ohio State University. Institutional Animal Care and Use Committee (IACUC) approved this study; nu/nu mice were purchased from The Jackson Laboratory. Tumors were established by injecting 1×10^7^ A549 cells subcutaneously into the right flanks of 6 wk-old female nude (nu/nu) mice. Each group consisted of five mice; 24 h before injection, A549 cells were pre-infected at MOI50 with Ad*GFP* or at MOI5 with Ad*FHIT*. Paclitaxel was administrated intravenously as a single treatment at the concentration of 40 mg/kg. Three days after treatment, mice were sacrificed and tumor weight assessed.

### Statistics

All graph values represent means ± SEM from three independent experiments with each measured in triplicate. The differences between two groups were analyzed with unpaired two-tailed Student's *t* test. P<0.05 was considered statistically significant and indicated with asterisks as described in figure legends.

The synergism of the combinations in this work were calculated using the chou-Talalay method. The resulting combination index (CI) offers quantitative definition for additive effect (CI = 1), synergism (CI<1), and antagonism (CI>1) in drug combinations.

## Supporting Information

Figure S1 Fhit and Annexin 4 protein expression was evaluated in a panel of cancer cell lines.
(TIF)
