# Supplementary figures and images for: Fhit Delocalizes Annexin A4 from Plasma Membrane to Cytosol and Sensitizes Lung Cancer Cells to Paclitaxel
Source: PLoS One. 2013 Nov 6;8(11):e78610. doi: 10.1371/journal.pone.0078610 (PMC3819369; doi:10.1371/journal.pone.0078610)

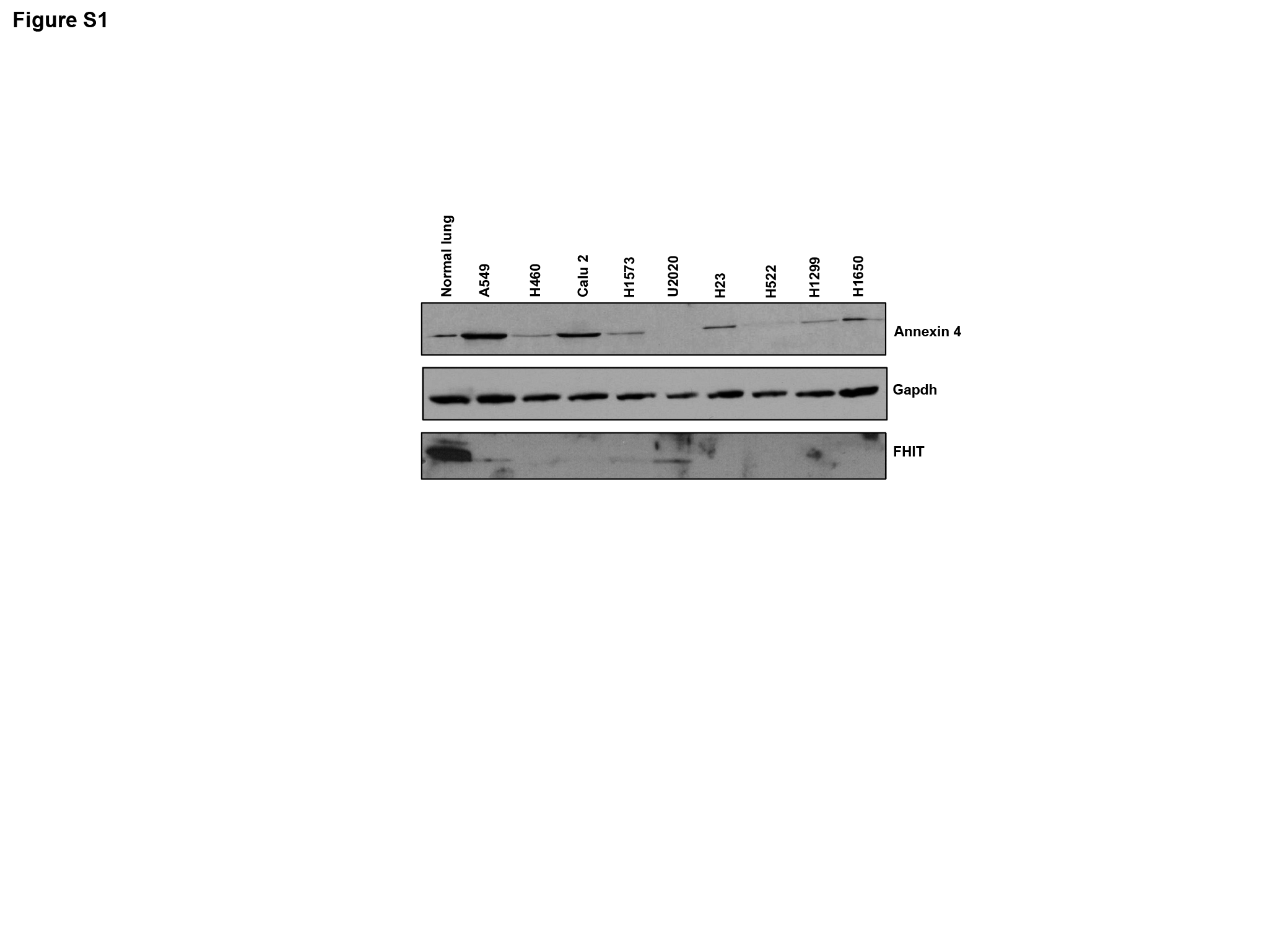

Supplement: Figure S1 — Fhit and Annexin 4 protein expression was evaluated in a panel of cancer cell lines. (TIF) [file pone.0078610.s001.tif]
